# Leucine-rich diet alters the ^1^H-NMR based metabolomic profile without changing the Walker-256 tumour mass in rats

**DOI:** 10.1186/s12885-016-2811-2

**Published:** 2016-10-03

**Authors:** Laís Rosa Viana, Rafael Canevarolo, Anna Caroline Perina Luiz, Raquel Frias Soares, Camila Lubaczeuski, Ana Carolina de Mattos Zeri, Maria Cristina Cintra Gomes-Marcondes

**Affiliations:** 1 Department of Structural and Functional Biology, Laboratory of Nutrition and Cancer, Institute of Biology, University of Campinas–UNICAMP, Campinas, 13083862 São Paulo Brazil; 2 Brazilian Biosciences National Laboratory, Campinas, São Paulo Brazil

**Keywords:** Cancer cachexia, Leucine supplementation, Metabolomic, Metabolic derangements, Walker 256 tumour

## Abstract

**Background:**

Cachexia is one of the most important causes of cancer-related death. Supplementation with branched-chain amino acids, particularly leucine, has been used to minimise loss of muscle tissue, although few studies have examined the effect of this type of nutritional supplementation on the metabolism of the tumour-bearing host. Therefore, the present study evaluated whether a leucine-rich diet affects metabolomic derangements in serum and tumour tissues in tumour-bearing Walker-256 rats (providing an experimental model of cachexia).

**Methods:**

After 21 days feeding Wistar female rats a leucine-rich diet, distributed in L-leucine and LW-leucine Walker-256 tumour-bearing groups, we examined the metabolomic profile of serum and tumour tissue samples and compared them with samples from tumour-bearing rats fed a normal protein diet (C – control; W – tumour-bearing groups). We utilised ^1^H-NMR as a means to study the serum and tumour metabolomic profile, tumour proliferation and tumour protein synthesis pathway.

**Results:**

Among the 58 serum metabolites examined, we found that 12 were altered in the tumour-bearing group, reflecting an increase in activity of some metabolic pathways related to energy production, which diverted many nutrients toward tumour growth. Despite displaying increased tumour cell activity (i.e., higher Ki-67 and mTOR expression), there were no differences in tumour mass associated with changes in 23 metabolites (resulting from valine, leucine and isoleucine synthesis and degradation, and from the synthesis and degradation of ketone bodies) in the leucine-tumour group. This result suggests that the majority of nutrients were used for host maintenance.

**Conclusion:**

A leucine rich-diet, largely used to prevent skeletal muscle loss, did not affect Walker 256 tumour growth and led to metabolomic alterations that may partially explain the positive effects of leucine for the whole tumour-bearing host.

## Background

Cancer is a worldwide health problem associated with an increasing number of deaths every year. Cachexia is one of the leading causes of death in cancer patients, accounting for nearly 30 % of such cases [1–3], and is a complex metabolic and nutritional syndrome characterised by involuntary weight loss that is mainly due to the wasting of skeletal muscle tissue. This muscle loss is also accompanied by adipose tissue loss, weakness affecting patient functional status and impairment of the immune system, which ultimately lead to a very poor quality of life and impaired host response to treatment [2, 4, 5].

Cancer cachexia also leads to metabolic derangements, and an increasing number of studies are emerging that examine altered metabolite profiles associated with various diseases, especially for cancer-associated cachexia [6]. Given that metabolites are excellent biomarkers, the presence and quantity of specific metabolites may provide a better understanding of cancer cell biology [7, 8]. For example, Der-Torossian and colleagues [7] described the changes between cachectic and non-cachectic gastrocnemius muscle tissue from C26 tumour-bearing mice and found that the glycolytic pathway was markedly altered from that of healthy mice. Additionally, Shen and colleagues [8] reported potential biomarkers in the urine of Walker-256 tumour-bearing rats during cancer progression, hypothesising that this alteration might have resulted from elevated cell proliferation, a reduction in the ß-oxidation of fatty acids and poor renal tubular reabsorption. The use of metabolomic science importantly permits a global understanding of biochemical processes and cellular states, reflecting changes in phenotype and also in cellular or tissue function [6, 9, 10]. The identities, concentrations and fluxes of metabolites are the final product of interactions between gene expression, protein expression and the cellular environment [11] and can therefore serve as indicators of the overall physiological status of patients [12].

Because cancer cachexia promotes metabolic alterations that lead to poor quality of life, it is imperative to increase the number of studies on and treatments for cachexia to improve patient care. One promising area of research is related to the use of nutritional supplementation to counteract physical changes accompanying disease [13]. For example, supplementation with the branched-chain amino acid has been shown to contribute to improved skeletal muscle mass that is diminished with ageing or due to diseases such as AIDS and diabetes [14]. Indeed, leucine is known to play an important role in skeletal muscle metabolism and regulates protein synthesis in following food intake, stimulating the mTOR pathway and inhibiting the ubiquitin-proteasome pathway [15, 16]. Leucine alone as well as a complete branched-chain amino acid mixture can further stimulate protein synthesis and decrease protein proteolysis [17]. Furthermore, previous studies from our group have shown that a leucine-rich diet can improve nitrogen balance and lean body mass [14, 18–20], specifically the skeletal muscle [18, 21–30], placental and heart [31] tissues in Walker 256 tumour-bearing rats. Thus, leucine supplementation may also be promising for the treatment and even prevention of cancer cachexia. Even still, while the role of leucine in stimulating skeletal muscle protein synthesis is well established in the literature [15, 17, 21, 32, 33], to date no study has evaluated the leucine-induced modulation on metabolomic profile in tumour-bearing hosts. In the present study, we develop a ^1^H NMR metabolomic profile (serum and tumour tissue) to evaluate the therapeutic effect of a leucine-rich diet in rats bearing Walker 256 tumours, which offer an experimental model of cachexia [34]. In this way, we are able to evaluate the metabolic derangements caused by tumour growth, and such knowledge may optimise the ability to treat changes in molecular and biochemical pathways that result from conditions such as cachexia.

## Methods

### Animals and diet

Female Wistar rats (approximately 90 ± 10 days old, obtained from the Animal Facilities at the State University of Campinas, UNICAMP, Brazil) weighing approximately 265 ± 10 g were housed in collective cages under controlled environmental conditions (light and dark 12/12 h; temperature 22 ± 2 °C; and humidity 50-60 %). The animals were monitored daily, weighed 3 times/week and received food and water *ad libitum*. Semi-purified diets were constructed in accordance with American Institute of Nutrition (AIN-93; [35]) while the leucine-supplemented diet was enriched with 3 % L-Leucine as in our previous works [14, 21, 24]. Both diets (control, C and leucine, L) contained similar amounts of nitrogen (approximately 2.84 g N_2_/100 g diet) for a protein content of approximately 18 %. All components of the diets are presented in Table 1.

**Table 1:** Diet components

	Diets	
	Control	Leucine
Components	(%)	(%)
Cornstarch^a^	39.7	38.7
Casein	20.0	20.0
Dextrin	13.2	12.2
Sugar	10.0	9.0
Fibre (cellulose micro fibre)	5.0	5.0
Salt mix	3.5	3.5
Vitamin mix	1.0	1.0
Cysteine	0.3	0.3
Choline	0.25	0.25
Fat (soy oil)	7.0	7.0
L-leucine ^b^	0.0	3.0

^a^Provided by Ingredion Products Brazil, ^b^Provided by Ajinomoto Interamericana Ind. & Com. Ltda. The diets contained similar amounts of nitrogen (approximately 13.2 mg N_2_/100 g food). The caloric adjustment of the amino acid-rich diet was accomplished by reducing the equivalent amount of carbohydrates that corresponded to isocaloric diets. The other ingredients contained the same amount of fat, fibre, salt and vitamin mix and cysteine and choline as the normo-protein diet

### Walker 256 tumour inoculation

This study employed the Walker 256 tumour, which is widely used as an experimental model of cancer cachexia syndrome. Walker 256 carcinoma cells (2.5 × 10^6^ viable cells) were injected subcutaneously into the right flank of the experimental rats on the first day of the experiment. The general guidelines of the UKCCCR (United Kingdom Co-ordinating Committee on Cancer Research, 1998) [36] regarding animal welfare were followed, and the experimental protocols were approved by the Institutional Committee for Ethics in Animal Research (CEEA/IB/UNICAMP, protocol # 2677-1).

### Experimental protocol

Thirty-five animals were randomly distributed into four experimental groups according to tumour implant status and nutritional leucine supplementation: two groups were fed a control diet (18 % protein): C, control group (*n* = 9) and W, Walker 256 tumour-bearing group (*n* = 9), while the other two other groups were fed a leucine-rich diet (18 % protein + 3 % leucine): L, leucine control group (*n* = 8) and LW, leucine Walker 256 tumour-bearing group (*n* = 9). All rats were monitored and weighed 3 times/week. At the end of the nutritional supplementation period, i.e., 21 days after tumour evolution, the animals were sacrificed without an overnight fast, their blood was collected, and tumours were resected and weighed. Blood samples were centrifuged at 1000 × g at 4 °C for 10 min and serum was stored at −20 °C for metabolomic analyses. The tumour tissue samples were frozen directly in liquid nitrogen and stored at −80 °C for metabolomic assays, western blotting and immunochemistry.

### Metabolomic analysis

#### Sample preparation for NMR analysis

Plasma samples were filtered through a Microcon YM-3 column (Amicon Ultra 0.5 mL, Sigma-Aldrich) with a 3-kDa membrane centrifuge filter for serum recovery (at 4 °C). Serum (0.2 mL) was diluted in an aqueous solution (0.6 mL) containing 10 % (v/v) deuterium oxide (D_2_O, 99.9 %; Cambridge Isotope Laboratories Inc., Massachusetts, USA), phosphate buffer (0.1 M, pH 7.4) and 0.5 mM TMSP-d4 (3-(trimethylsilyl)-2,2',3,3'-tetradeuteropropionic acid from Sigma-Aldrich), then transferred to a 5-mm NMR tube (Norell Standard Series 5 mm, Sigma-Aldrich) for immediate acquisition.

Tumour samples were processed following Le Belle and colleagues’ protocol [37]. Briefly, tumour tissue fragments were weighed, added to a cold methanol/chloroform solution (2:1 v/v, total of 2.5 mL) and sonicated (VCX 500, Vibra-Cell, Sonics & Material Inc., USA) for 3 min with a 10-s pause interval between each minute. A cold chloroform/distilled water solution (1:1 v/v, total of 2.5 mL) was then added to the samples. Samples were briefly vortexed (to form an emulsion) and centrifuged at 3.1 × 10^3^ g for 20 min at 4 °C. The upper phase (containing methanol, water and polar metabolites) was collected and dried in a vacuum concentrator (miVac Duo Concentrator, GeneVac, UK). The remaining solid phase was rehydrated in 0.6 mL of D_2_O-containing phosphate buffer (0.1 M, pH 7.4) and 0.5 mM of TMSP-d4. Samples were added to a 5-mm NMR tube for immediate acquisition.

#### NMR data acquisition and metabolite identification

^1^H NMR spectra of samples were acquired using a Varian Inova NMR spectrometer (Agilent Technologies Inc., Santa Clara, USA) equipped with a triple resonance probe and operating at a ^1^H resonance frequency of 500 MHz and constant temperature of 298 K (25 °C). A total of 256 free induction decays were collected with 32-k data points over a spectral width of 16 ppm. A 1.5-s relaxation delay was incorporated between scans, during which a continual water presaturation radio frequency (RF) field was applied. Spectral phase and baseline corrections, as well as the identification and quantification of metabolites present in samples, were performed using Chenomx NMR Suite 7.6 software (Chenomx Inc., Edmonton, Canada).

### Tumour immunohistochemistry for tumour Ki-67 and vessel number

Fragments of tumour tissue were fixed for 24 h in 4 % paraformaldehyde solution before being embedded in paraffin. From each tissue sample, 5-μm sections were selected for the Ki-67 immunoperoxidase reaction. For the immunohistochemistry assay, the paraffin was removed. For antigen retrieval, the sections were incubated with 10 mM sodium citrate buffer (pH 6) for 1 h at 80 °C, washed with 0.05 M Tris-buffered saline (TBS, pH 7.4) and incubated with TBS containing 0.3 % H_2_O_2_ for endogenous peroxidase activity blockade. The sections were then permeabilised for 1 h with 0.1 % Tween® 20 and 5 % of fat-free milk in TBS. The sections were then incubated with a rabbit monoclonal anti-Ki67 (1:50; Spring Bioscience, Pleasanton, CA) antibody at 4 °C overnight and, after this period, incubated with anti-rabbit for rat tissues (Simples Stain Rat Max Po; N-Histofine®; Nichirei Biosciences inc., Tokyo, Japan) for 1.5 h. The positive proliferating cells were detected with 3,3'-diaminobenzidine (DAB; Sigma- Aldrich Chemicals, St Louis, MO, USA) solution (10 % DAB and 0.2 % H_2_O_2_ in TBS). Finally, the sections were stained with Ehrlich’s haematoxylin and mounted for microscopy. The Ki67-positive cells were counted using Image-Pro Plus software after capturing the image on a Leica microscope using 100× magnification. For negative controls, tumour sections from each group were incubated in PBS without the first antibody and then incubated with the biotinylated anti-goat secondary antibody followed by reaction with DAB, as described above. The number of positive cells and vessel number were determined by counting 5 fields (500 μm^2^ each) in one slide from each of at least six rats per group.

### Tumour western blotting

Tumour tissue samples were homogenised in protein extraction buffer (20 mM N-2-hydroxy ethylpiperazine-N-2-ethanesulfonic acid, 100 mM KCl, 0.2 mM EDTA, 2 mM EGTA, 1 mM dithiothreitol, 50 mM NaF, 1 mM DAB tetrahydrochloride, 0.5 mM orthovanadate and 50 mM glycine, pH 7.4) followed by centrifugation at 10,000 × g for 15 min at 4°C and were then separated by 10 % SDS–PAGE electrophoresis under reducing conditions. After gel electrophoresis and protein transference onto a nitrocellulose membrane, the proteins were blocked at room temperature for 1 h in 5 % non-fat dry milk. The membranes were then incubated overnight at 4 °C with antibodies against mTOR (Cell Signalling; diluted 1:1000). Immunoreactivity was detected by the sequential incubation of membranes with anti-mouse secondary antibody for 1 h at room temperature, which was visualised using a chemiluminescence detection system. The level of mTOR was estimated versus the constant level of the 50 kDa protein α-tubulin (Cell Signalling; diluted 1:1000).

### Statistical analyses

Results are shown as the mean ± standard deviation, after analysis of all data by Graph Pad Prism 6.0 software (Graph-Pad Software, Inc). For comparisons among multiple groups (e.g., C, W, L and LW), data were evaluated with analysis of variance (two-way ANOVA) followed by post-hoc comparison using Bonferroni’s test [38]. For direct comparison between the two groups (e.g., analysis of the tumour tissue in the W and LW groups), the data were analysed using Student’s t-test. A significant difference was indicated for *P* ≤ 0.05. Metabolite set enrichment analysis (MSEA) was performed to determine the metabolic pathways impacted with the changed metabolites among experimental groups. MSEA was conducted using the MetaboAnalyst 3.0 tool, and a significant difference was indicated for *P* ≤ 0.05 [39].

## Results

### Walker 256 tumour induced cachexia in both tumour-bearing groups

Both tumour-bearing groups (W and LW) exhibited a decrease in the rat carcass weight, a tumour weight to body weight ratio higher than 10 % and a reduction in serum albumin concentration (Table 2). Leucine supplementation also leads to a lower cachexia index in the LW group when compared with the W group. Under our experimental conditions, this reduction trended to reach significance with *P* = 0.0561 (Table 2).

**Table 2:** Morphometric parameters and cachexia indicators ^(a)^

	C (*n* = 9)	W (*n* = 9)	L (*n* = 8)	LW (*n* = 9)
Morphometric parameters				
Initial body weight (g)	253.4 ± 23.5	264.9 ± 14.6	249.4 ± 24.7	257.0 ± 16.5
Carcass weight (g)	247.5 ± 29.8	184.5 ± 33.7^*, **^	243.8 ± 24.7	188.6 ± 21.8^*, **^
Cachexia index ^(a)^	-	63.9 ± 2.6	-	54.8 ± 2.9 ^***^
Tumour weight (g)	-	36.04 ± 10.2	-	36.82 ± 9.0
Relative tumour weight (%)^(b)^	-	14.79 ± 4.7	-	15.20 ± 4.8
Serum albumin (g/dL)	2.6 ± 0.2	1.5 ± 0.1^*^	2.7 ± 0.3	1.6 ± 0.2^*, **^

Data are expressed as the mean ± SD. Legend: C control; W, tumour-bearing (fed with control diet, 18 % protein); L, control; LW, tumour-bearing (fed with leucine-rich diet, 18 % protein + 3 % leucine). Carcass weight represents the body weight without the weight of the gastrointestinal tract, liver, muscles and tumour. ^(a)^Cachexia index = [(initial body mass – carcass mass + tumour weight + body mass gain of control)/(initial body mass + body mass gain of control)] × 100 %. [1]. ^(b)^Relative tumour weight corresponds to the ratio of tumour and body weights, expressed as a percentage. ^*^ *P* ≤ 0.05 in comparison with the C group; ^**^ *P* ≤ 0.05 in comparison with the L group; ^***^ *P* = 0.0561 in comparison to the W group

**Fig. 1:**
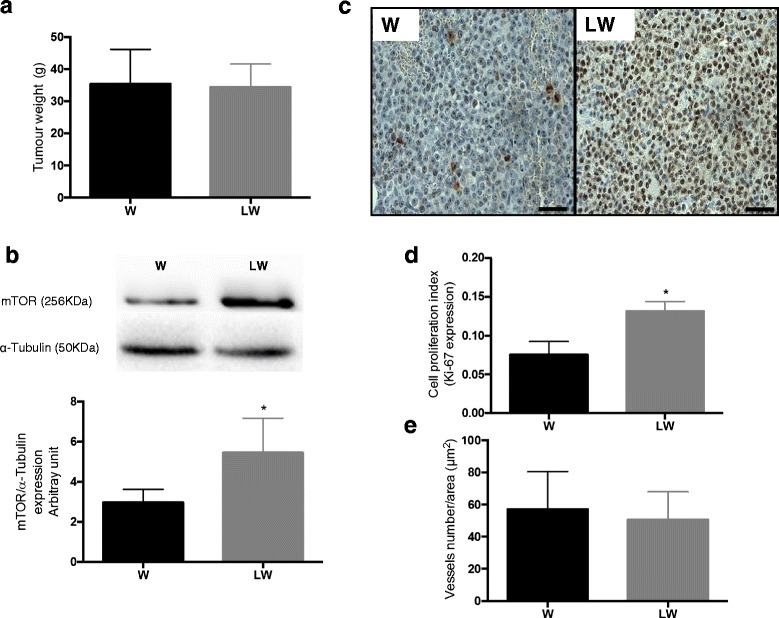
Tumour parameters. **a** Tumour weight (g), **b** mTOR (Western Blot images represent the best results from 6 animals per group), **c**: Immunohistochemistry image for Ki-67 protein (magnification 200×), **d**: K﻿i-67 expression and **e**: Number of vessels. For details, see Methods. The graphics express the results as the mean ± SD.* *P* ≤ 0.05 for comparison with the W group

Tumour weight and vessel number did not differ between the W and LW groups (Table 2 and Fig. 1a and e), even though the tumour tissue of the LW group showed an increase in mTOR and Ki-67 protein expression in comparison to W group (Fig. 1b, c and d).

### Serum metabolomic alterations

In our ^1^H NMR metabolomics analysis system, which mainly targeted water-soluble/polar metabolites, we detected 58 metabolites in serum samples for all four experimental groups. Among these metabolites, only 3 were exclusive to tumour-bearing rats: 3-Methyl-2-oxovalerate, 4-Hydroxyphenyl Lactate and 3-Methylhistidine (Table 3).

**Table 3:** Serum metabolic concentration (μM)

Metabolites	C (average ± SD)	W (average ± SD)	L (average ± SD)	LW (average ± SD)
α-Aminobutyrate	5.8 ± 4.8	7.9 ± 1.8	4.5 ± 1.6	10.9 ± 7.2
2-Hydroxybutyrate	3.3 ± 2.8	24.7 ± 7.0	2.7 ± 0.8	32.5 ± 22.0 ^*, ***^
2-Hydroxyisovalerate	0.9 ± 0.1	18.8 ± 5.7	1.0 ± 0.5	33.3 ± 25.1 ^*, ***^
2-Oxoglutarate	5.4 ± 1.0	7.1 ± 1.1	5.1 ± 1.6	7.5 ± 2.5
2-Oxoisocaproate	0.6 ± 0.1	2.9 ± 0.9 ^*^	0.9 ± 0.2	2.7 ± 0.5 ^*, ***^
β-Hydroxybutyrate	14.5 ± 5.5	150.4 ± 70.3	22.9 ± 5.5	480.7 ± 278.3 ^*, **, ***^
3-Hydroxyisobutyrate	8.6 ± 2.2	21.1 ± 6.5	7.0 ± 2.0	32.5 ± 17.5 ^*, ***^
3-Methyl-2-oxovalerate	0.0 ± 0.0	4.4 ± 1.4 ^*^	0.0 ± 0.0	3.4 ± 1.1 ^*, ***^
4-Hydroxyphenyllactate	0.0 ± 0.0	2.8 ± 0.6 ^*^	0.0 ± 0.0	6.8 ± 4.6 ^*, **, ***^
Acetate	12.2 ± 2.1	33.3 ± 19.5	15.0 ± 4.5	35.0 ± 17.6
Acetoacetate	0.9 ± 0.4	3.4 ± 1.6	1.1 ± 0.4	7.6 ± 3.9 ^*, **, ***^
Acetone	5.0 ± 0.7	61.6 ± 19.2 ^*^	4.3 ± 1.6	53.3 ± 2.9 ^*, ***^
Alanine	250.6 ± 33.4	477.5 ± 182.1	220.2 ± 53.3	419.9 ± 236.4
Allantoin	25.0 ± 2.5	132.2 ± 43.3 ^*^	18.8 ± 14.6	156.4 ± 68.4 ^*, ***^
Arginine	60.4 ± 2.1	37.4 ± 7.9 ^*^	53.0 ± 16.3	38.9 ± 16.4
Asparagine	25.8 ± 1.7	18.7 ± 5.8	20.6 ± 5.4	19.4 ± 2.8
Aspartate	14.2 ± 1.6	12.2 ± 2.9	13.4 ± 4.2	18.1 ± 5.9
Betaine	34.9 ± 9.6	119.7 ± 61.0	30.9 ± 8.4	132.3 ± 86.1 ^*, ***^
Carnitine	12.2 ± 1.5	15.3 ± 4.3	9.8 ± 3.2	17.7 ± 8.1
Choline	9.2 ± 2.0	16.2 ± 4.1	8.5 ± 2.5	20.4 ± 11.0
Citrate	70.8 ± 7.7	105.1 ± 22.1	57.3 ± 12.1	190.9 ± 112.2 ^***^
Creatine	87.1 ± 7.9	188.0 ± 43.0	73.4 ± 18.7	252.5 ± 138.1 ^*, ***^
Creatinine	9.6 ± 0.8	12.7 ± 4.0	6.7 ± 2.6	21.7 ± 9.8
Dimethylamine	0.5 ± 0.2	2.2 ± 0.6	0.6 ± 0.1	4.6 ± 2.7 ^*, ***^
Ethanol	874.3 ± 858.2	2159.4 ± 1079.9	949.5 ± 816.4	1257.7 ± 109.2
Formate	13.3 ± 8.4	31.0 ± 13.4	12.5 ± 1.8	27.9 ± 17.1
Fumarate	2.0 ± 0.3	1.3 ± 0.2	1.6 ± 0.3	1.4 ± 0.9
Glucose	1502.8 ± 171.0	708.7 ± 407.2 ^*^	1305.1 ± 531.3	679.0 ± 479.3 ^*^
Glutamate	68.7 ± 14.8	73.0 ± 15.8	71.1 ± 18.0	83.4 ± 34.6
Glutamine	258.3 ± 18.1	110.5 ± 33.0 ^*^	224.7 ± 65.1	105.7 ± 45.5 ^*, ***^
Glycerol	348.2 ± 61.1	266.4 ± 151.7	287.7 ± 53.5	193.9 ± 119.3
Glycine	80.2 ± 7.5	120.2 ± 37.2	67.0 ± 17.9	117.6 ± 31.6
Histidine	23.7 ± 2.6	24.2 ± 5.5	17.8 ± 4.2	24.7 ± 7.1
Isoleucine	31.7 ± 3.0	24.9 ± 4.9	21.9 ± 6.3	25.6 ± 10.7
Lactate	4201.1 ± 305.4	5007.8 ± 599.1	4133.7 ± 1165.8	4242.8 ± 684.4 ^**^
Leucine	52.4 ± 5.5	43.5 ± 5.5	77.1 ± 13.6 ^*^	59.7 ± 20.0
Lysine	236.6 ± 49.3	178.4 ± 55.7	204.9 ± 61.9	145.7 ± 59.3
Methionine	25.8 ± 5.2	22.3 ± 3.7	22.2 ± 6.0	19.3 ± 4.9
Dimethylglycine	2.3 ± 0.6	5.8 ± 2.4	1.9 ± 0.6	14.6 ± 11.9
O-Acetylcarnitine	4.7 ± 1.1	12.0 ± 2.8	3.7 ± 1.1	14.9 ± 6.7 ^*, ***^
Ornithine	9.2 ± 0.8	13.7 ± 5.3	8.3 ± 3.2	22.9 ± 16.2
Pantothenate	1.8 ± 0.2	3.0 ± 1.0	1.9 ± 0.4	4.2 ± 2.9
Phenylalanine	21.0 ± 2.9	34.3 ± 5.9	18.8 ± 5.3	32.5 ± 12.0
Proline	113.5 ± 33.3	155.7 ± 60.6	96.7 ± 35.7	123.3 ± 50.4
Pyruvate	68.9 ± 17.5	76.3 ± 31.9	63.1 ± 25.2	71.6 ± 20.0
Sarcosine	0.7 ± 0.1	2.9 ± 0.4 ^*^	0.8 ± 0.5	2.6 ± 1.2 ^*, ***^
Serine	97.3 ± 3.8	46.6 ± 16.0 ^*^	77.8 ± 30.4	43.4 ± 17.6 ^*, ***^
Succinate	37.8 ± 5.5	36.7 ± 14.3	42.4 ± 23.1	52.2 ± 44.5
Taurine	249.0 ± 32.7	227.2 ± 44.6	205.5 ± 71.3	261.8 ± 142.7
Threonine	178.3 ± 40.8	77.4 ± 29.3 ^*^	166.6 ± 77.2	65.3 ± 27.3 ^*, ***^
Tryptophan	1.5 ± 0.3	10.9 ± 2.8 ^*^	1.6 ± 0.7	6.0 ± 2.7 ^*, **, ***^
Tyrosine	31.1 ± 6.0	47.2 ± 9.4	22.3 ± 6.1	38.6 ± 18.6
Uracil	4.1 ± 1.1	10.0 ± 3.8	4.5 ± 0.9	9.1 ± 4.2
Urea	522.7 ± 92.1	1022 ± 400.3	492.2 ± 136.5	2701.1 ± 1817.3 ^*, **, ***^
Valine	75.1 ± 6.8	51.5 ± 10.4	50.9 ± 13.6	52.8 ± 19.5
Myoinositol	22.7 ± 1.7	98.0 ± 11.0	21.6 ± 6.4	153.6 ± 88.3 ^*, ***^
sn-Glycerol-3-phosphocholine	3.3 ± 0.8	5.7 ± 1.4	3.4 ± 0.6	6.2 ± 4.3
3-Methylhistidine	0.0 ± 0.0	12.3 ± 4.4 ^*^	0.0 ± 0.0	17.9 ± 10.3 ^*, ***^

Data are expressed as the mean ± SD. For details, see the Methods and Results sections ^*^ *P* ≤ 0.05 in comparison with the C group; ^**^ *P* ≤ 0.05 in comparison with the W group and ^***^ *P* ≤ 0.05 in comparison with the L group

### Leucine was the only changed metabolite in the serum of healthy animals fed a leucine-rich diet

**Fig. 2:**
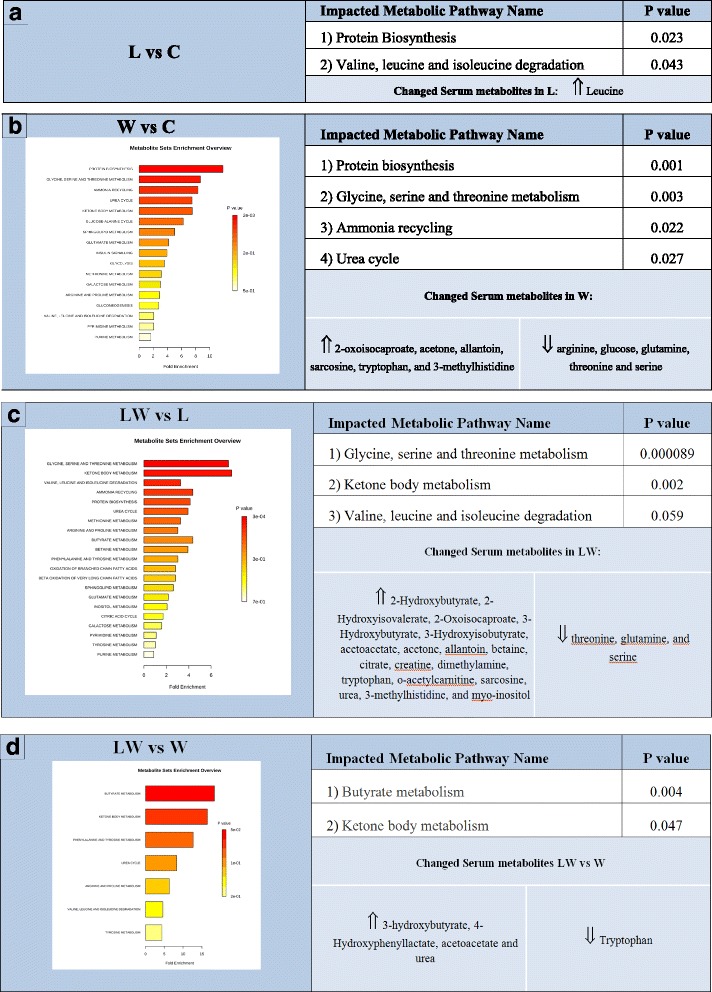
Impacted metabolic pathways and changed metabolites in tumour-bearing rats (W and LW groups) compared to non-tumour-bearing animals (C and L groups). **a** Summary of significantly impacted pathways (*P* ≤ 0.05) analysed by the different metabolites found in the leucine group in comparison to the C group. **b** Comparison of tumour-bearing rats fed with normal diet (W) and the C group with a serum list of increased and decreased metabolites levels in W group. Metabolite set enrichment analysis revealed the affected pathways. **c** Comparison of tumour-bearing rats (LW) and non-tumour-bearing rats (L) fed a leucine-rich diet with a list of serum metabolites, which both increased and decreased in the LW group serum. Metabolite set enrichment analysis revealed the affected pathways. **d** Comparison between both tumour-bearing rats fed a normal diet (W) and a leucine-rich diet (LW) with a list of metabolites that increased and decreased in the LW serum compared to the W serum. All data were processed using the *Metaboanalyst* tool [39]. For details, see Methods

In order to analyse the modulatory effect of nutritional supplementation with leucine, we first analysed both control groups (C and L) and found that leucine was the only metabolite that increased in the L group in comparison with the C group (Table 3; Fig. 2a).

### Walker 256 tumour growth induces a variety of changes in metabolomic serum profile

A comparison of the tumour-bearing (W) and control (C) groups showed changes in 12 metabolites (21.8 %), demonstrating that the cancer cachexia severely affected metabolism in the whole body (Table 3, Fig. 2b). Moreover, levels of the metabolites 2-oxoisocaproate, acetone, allantoin, sarcosine, tryptophan and 3-methylhistidine increased in the W group relative to the C group while arginine, glucose, glutamine, threonine and serine levels decreased relative to the C group (Fig. 2b). With these alterations in serum metabolite levels, we found that four metabolic pathways were significantly impacted (*P* ≤ 0.05) due to the evolution of the Walker 256 tumour (Fig. 2b), namely protein biosynthesis, glycine, serine and threonine metabolism, ammonia recycling and the urea cycle.

### Leucine-rich diet modulated the tumour-induced changes in serum metabolomic profile

Tumour-bearing rats fed a leucine-rich diet (LW) showed alterations in 23 (39.6 %) serum metabolites in comparison to the control group (L) (Table 3 and Fig. 2c). Among these metabolites, the levels of the following 16 were increased for LW when compared with the L group: α-hydroxybutyrate, 2-hydroxyisovalerate, 2-oxoisocaproate, β-hydroxybutyrate, 3-hydroxyisobutyrate, acetoacetate, acetone, allantoin, betaine, citrate, creatine, dimethylamine, tryptophan, o-acetylcarnitine, sarcosine, urea, 3-methylhistidine and myoinositol. Only three metabolites decreased in the LW group: threonine, glutamine and serine. We also observed that three main pathways (*P* ≤ 0.05) were impacted by Walker 256 tumour evolution under a leucine-rich diet, namely glycine, serine and threonine metabolism, ketone body metabolism and valine, leucine and isoleucine degradation (Fig. 2c).

### The leucine-rich diet modulated the impacted pathway seen in tumour-bearing rats, leading to an increase in the synthesis and degradation of ketone bodies

**Fig. 3:**
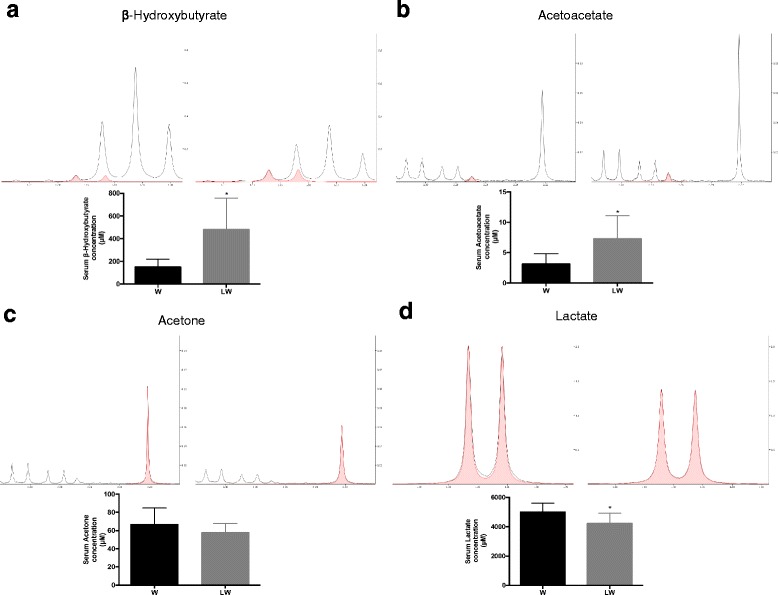
The most significant metabolites changed in both tumour-bearing groups. **a** Region of 600 MHz liquid ^1^H NMR spectra showing β-hydroxybutyrate metabolite in the serum from W and LW groups. **b** Region of the 600 MHz liquid ^1^H NMR spectra showing the acetoacetate metabolite. **c** Region of 600 MHz liquid ^1^H NMR spectra of acetone metabolite. **d** Region of 600 MHz liquid ^1^H NMR spectra showing lactate metabolite in the serum of tumour-bearing rats. The graphics express the results obtained from the area under the curve of spectral regions and are expressed as the mean ± SD.* *P* ≤ 0.05 for comparison with the W group. For details, see Methods

In order to evaluate the effect of the leucine-rich diet in tumour-bearing rats, we compared the W and LW groups. In the LW group, we found increased metabolites, such as β-hydroxybutyrate (Fig. 3a), 4-hydroxyphenyllactate, acetoacetate (Fig. 3b) and urea, relative to the W group (Table 3). Tryptophan and lactate levels also decreased in the LW group compared to the W group (Table 3; Fig. 3c and d). Analysing these changed metabolites, we observed two main impacted pathways in LW groups namely butyrate metabolism and ketone body metabolism (Fig. 2d).

### Metabolomic profile of Walker 256 tumour tissue

**Fig. 4:**
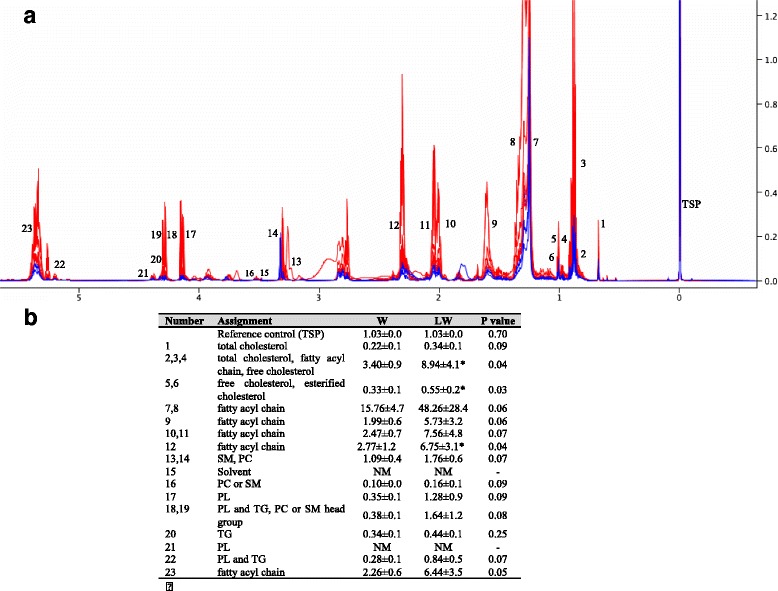
**a** ^1^H NMR spectrum of apolar tumour tissue metabolites extracted with chloroform from W and LW groups. **b** Table describing the apolar metabolites found in both tumour-bearing groups. The identified numbers in the spectrum are described in the table attached to the figure. Legend: W, tumour-bearing (*blue lines*); LW, leucine-treated tumour-bearing group (*red lines*). The results are expressed as the mean ± SD.* *P* ≤ 0.05 for comparison with the W group. For details, see Methods

Our ^1^H-NMR metabolomic analysis system largely targeted water-soluble metabolites (methanol phase), and 69 metabolites in total were detected in tumour tissue samples (Table 4). We also evaluated the non-water-soluble metabolites (lipids) present in the chloroform phase (Fig. 4). In order to assess the effect of the leucine-rich diet on tumour metabolism, we compared metabolites present in tumour tissue from the W and LW groups. Of the 69 water-soluble metabolites, only glycerol differed between the two groups and was found a decrease for LW in comparison to W (Table 4). The non-water-soluble phase revealed that the tumours of the animals fed with a leucine-rich diet had increased lipid deposits, with substantial differences between both groups. In particular, the LW group exhibited increased values (*P* ≤ 0.05) of cholesterol and a fatty acyl chain in comparison to the W group (Fig. 4).

**Table 4:** Tumour tissue metabolic concentration (μM)

Metabolites	W (average ± SD)	LW (average ± SD)
2-Aminobutyrate	16.9 ± 7.3	13.0 ± 5.7
2-Hydroxybutyrate	12.3 ± 4.1	10.4 ± 5.1
2-Hydroxyisovalerate	6.6 ± 2.6	5.4 ± 3.1
3-Hydroxybutyrate	52.9 ± 47.0	49.7 ± 37.3
3-Hydroxyisobutyrate	6.1 ± 1.7	7.5 ± 2.8
4-Hydroxyphenyllactate	1.6 ± 0.5	2.0 ± 1.0
ADP	16.3 ± 4.3	17.8 ± 8.7
AMP	65.6 ± 39.9	77.3 ± 29.4
Acetate	62.5 ± 12.9	57.3 ± 22.3
Acetone	1.5 ± 1.0	1.6 ± 0.6
Alanine	889.6 ± 477.7	813.7 ± 377.2
Anserine	47.2 ± 29.1	28.2 ± 18.0
Ascorbate	91.3 ± 42.9	87.8 ± 40.5
Asparagine	78.9 ± 35.8	66.1 ± 22.9
Aspartate	106.8 ± 54.5	84.4 ± 40.2
Betaine	56.6 ± 33.8	50.2 ± 29.8
Carnitine	24.8 ± 14.1	22.8 ± 8.1
Choline	22.7 ± 6.2	20.5 ± 10.8
Creatine	118.7 ± 53.2	147.6 ± 82.4
Cytidine	13.0 ± 5.3	11.8 ± 5.7
Dimethylamine	2.2 ± 1.0	1.7 ± 1.4
Ethanol	3.3 ± 1.5	5.3 ± 2.7
Formate	13.4 ± 4.6	10.4 ± 5.4
Fumarate	7.2 ± 1.8	7.4 ± 2.5
Glucose	26.3 ± 11.5	31.1 ± 11.8
Glutamate	541.2 ± 210.0	536.0 ± 208.1
Glutamine	67.8 ± 49.3	54.5 ± 31.7
Glutathione	17.8 ± 8.4	19.6 ± 11.3
Glycerol	46.8 ± 10.6	35.0 ± 7.0*
Glycine	534.3 ± 302.2	466.5 ± 170.9
Guanosine	2.3 ± 1.0	2.0 ± 0.8
Histidine	39.0 ± 15.1	36.2 ± 13.5
Hypoxanthine	31.6 ± 9.0	23.7 ± 8.5
Inosine	61.3 ± 26.2	55.4 ± 23.8
Isoleucine	42.7 ± 16.7	35.0 ± 11.9
Lactate	3193.4 ± 1295.5	3390.1 ± 1713.1
Leucine	86.8 ± 30.2	102.9 ± 56.4
Lysine	168.6 ± 58.1	164.0 ± 76.5
Malate	81.2 ± 24.6	71.5 ± 31.6
Methionine	35.1 ± 16.4	24.5 ± 9.6
N, N-Dimethylglycine	1.6 ± 0.6	2.1 ± 0.8
Niacinamide	19.8 ± 6.2	16.8 ± 5.5
O-Acetylcarnitine	8.8 ± 1.8	8.9 ± 4.7
O-Phosphocholine	206.0 ± 86.2	219.2 ± 77.5
O-Phosphoethanolamine	516.4 ± 206.1	447.2 ± 194.8
Ornithine	9.5 ± 3.5	10.7 ± 3.0
Oxypurinol	669.6 ± 457.5	688.7 ± 433.6
Phenylalanine	52.1 ± 24.6	40.9 ± 13.9
Proline	264.1 ± 101.0	254.6 ± 106.7
Propionate	3.7 ± 1.4	4.2 ± 1.7
Sarcosine	4.5 ± 2.5	4.0 ± 2.4
Serine	129.6 ± 50.2	121.6 ± 56.6
Succinate	78.5 ± 42.3	84.9 ± 43.3
Taurine	750.7 ± 165.6	832.3 ± 297.0
Threonine	146.8 ± 60.3	190.9 ± 125.8
Tryptophan	18.6 ± 7.0	14.8 ± 5.1
Tyrosine	69.5 ± 35.5	50.4 ± 18.3
UDP-N-Acetylglucosamine	17.4 ± 7.9	15.1 ± 6.1
UDP-galactose	6.5 ± 2.2	6.7 ± 1.9
UDP-glucose	11.4 ± 2.7	15.2 ± 5.2
Uracil	25.5 ± 5.9	20.5 ± 6.5
Uridine	14.1 ± 5.0	12.9 ± 5.3
Valine	88.7 ± 33.4	75.9 ± 26.5
Myoinositol	81.4 ± 31.4	79.1 ± 40.5
sn-Glycero-3-phosphocholine	64.8 ± 33.8	80.4 ± 45.3
β-Alanine	16.6 ± 6.3	19.5 ± 5.3
3-Methylhistidine	16.6 ± 10.5	20.1 ± 16.6
τ-Methylhistidine	3.9 ± 1.2	5.4 ± 3.5

Data are expressed as the mean ± SD. For details, see the Methods and Results sections

*P ≤ 0.05 in comparison with the W group

## Discussion

The present work utilised ^1^H NMR to develop metabolomic profiles for all four rat groups to better understand the effect of leucine supplementation on tumour growth. Profound metabolic changes were observed in W and LW groups, especially related to protein and amino acid metabolism. These changes were likely associated with a cachexia state induced by an increase in protein degradation to support tumour growth. Both tumour-bearing groups also exhibited alterations in specific pathways related to the metabolism of glycine, serine, threonine, arginine and proline. These pathways might be involved in the high activity of tumour cells and specific host (e.g., muscle) tissues. Moreover, the altered metabolites are those that play a role in amino acid synthesis (aminoacyl t-RNA biosynthesis) [40], and these may likely provide newly synthesised amino acids for a different metabolic pathway, such as gluconeogenesis, or these amino acids could be directly used by the tumour tissue as an energy source. Increased body protein turnover is normally related to tumour growth [1, 5, 41], where decreased protein synthesis and increased protein degradation occur in response to tumour effects that mobilise muscle proteins. The nitrogen from muscle tissue is a source of building blocks for rapidly growing tumours such as the Walker 256 tumour [27, 34]. Indeed, high serum levels of 3-methylhistidine, a product of peptide bond synthesis and the methylation of actin and myosin, was detected in both tumour-bearing groups, and the corresponding quantity of 3-methylhistidine provides an index for the rate of muscle protein breakdown [42]. Researchers have previously shown a positive correlation between increased 3-methylhistidine and cancer progression, along with cancer cachexia, due to the high muscle protein breakdown [43]. In agreement with this result, we observed that serum levels of creatine and creatinine were elevated in both tumour-bearing groups relative to the control groups, and the metabolites 3-methylhistidine and creatine were even higher in LW group compared to W group (Table 3). Moreover, while those protein subproducts were elevated in LW group, this not reflected in cachexia index which trended to be lower in the LW group than in the W group (*P* = 0.0561). These results suggest that leucine supplementation may be capable of stimulating protein synthesis and, consequently, may lead to a positive protein net balance even amidst a high rate of protein degradation, as shown in our previous studies [44, 45]. The impacted metabolic pathways determined here also suggest that the leucine-rich group may divert the metabolism to improve protein synthesis and also utilised other substrates as energy sources. Furthermore, a significant increase in the tryptophan serum levels for the W group in comparison to LW group suggests that the consumption of a leucine-rich diet may be associated with a lower tryptophan serum content, correspondingly lower serotonin levels and thus a decreased anorexigenic effect [14, 21, 23, 24, 45] and cachexia-associated fatigue state [46].

Tumour cells require a large energy supply to grow and exhibit a special mechanism for nutrient uptake, preferentially utilising glucose and glutamine as energy sources [47]. Thus, as might be expected, our data revealed that serum glucose level decreased in both W and LW groups, and consequently the serum ketone body levels (β-hydroxybutyrate, acetone and acetoacetate) also increased in these groups. In addition to this low serum glucose content, the ketogenic metabolites phenylalanine and tyrosine [48, 49] likely contributed to the serum elevation of ketone bodies observed in both groups, although the elevation was more pronounced in the LW group (Table 3). This observation might be explained by considering that for metabolism in a glucose-poor environment, excess leucine could also act as a ketone precursor and promote elevated blood levels [48, 49]. The ketone bodies could accordingly provide additional energy to the LW skeletal muscle and host tissues that is not available to the W group. Besides acting as a fuel source to supply energy for cellular activity of various tissues, ketone bodies, especially acetoacetate (AA), can also promote muscle cell proliferation [50], probably justifying the benefits found in LW group. Recently, Zou and colleagues (2016) found a novel function to AA in promoting muscle cell proliferation. AA accelerates muscle regeneration and ameliorates muscle dystrophy, acting through activation of the MEK1-ERK1/2-cyclin D1 pathway, revealing a novel mechanism in which AA serves as a signalling metabolite in muscle cell function [50]. We note that there was an inexplicable (though not statistically significant) increase in acetone levels in the W group, which might also act as a source of enhanced ketogenesis [51]. While healthy cells, such as skeletal muscle cells, readily adapt to use ketones as an efficient energy substrate, some cancers cells do not exhibit this metabolic flexibility [52–54]. For instance, some neoplastic cells lack certain key mitochondrial enzymes and have thus a decreased ability to metabolise ketone bodies for energy production [53, 55–57]. In a study of Walker 256 tumour cells, Fearon and collaborators [58] measured the three major enzyme activities responsible for the metabolism of ketone bodies in the mitochondria and observed no activity of the enzyme 3-ketoacid-CoA transferase, aside from significant amounts of 3-hydroxybutyrate dehydrogenase and acetyl-CoA acetyltransferase. Our data revealed that even with the high availability of β-hydroxybutyrate and acetoacetate in serum from the LW group, the tumour size remained the same between W and LW groups. Thus, we hypothesise that mitochondrial enzymatic deficiency or some structural/functional mitochondrial damage likely impaired the ability of the Walker 256 tumour cells to metabolise ketone bodies as an extra energy source. Ongoing work in our laboratory is endeavouring to confirm and elucidate this proposed tumour cell deficiency. We note that in support of this argument, a recent study showed that when glioma-bearing rats were fed a ketogenic diet, tumour growth was unaffected even with a normal ketone body metabolism in RG2 and 9L gliomas cells and an upregulation of ketone body transport [59].

**Fig. 5:**
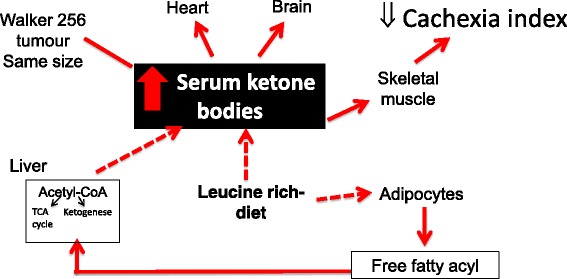
Proposed mechanism of leucine-rich diet leading to ketosis. TCA: tricarboxylic acid cycle

In spite of their tumours, the LW group was observed to maintain a serum leucine content, which was slightly elevated compared to the W group (Table 3). The absence of elevated of leucine levels in LW, as observed in the L group, may be partially explained by considering that leucine is a ketogenic amino acid. Thus, the most affected pathway in the tumour-bearing rats appears to be the synthesis of ketone bodies, and the excess leucine consumed by the LW group was rapidly deviated to this pathway (Fig. 5).

Most of the cancer cells exhibit a dysregulated metabolic phenotype characterised by lactate fermentation in the presence of oxygen, a phenomenon known as Warburg effect [60–62]. The conversion of glucose to pyruvate in neoplastic cells plays a major role in this rapid cellular growth, as it provides several intermediates required for biomass synthesis by routeing the carbon flux through the pentose phosphate pathway [47, 63, 64]. Furthermore, the conversion of pyruvate to lactate leads to acidosis in tumour microenvironments, which facilitates the invasion and metastasis of these cancer cells [65]. As previously observed in the literature, we found increased lactate serum levels in the W group in comparison to the control group, C. This lactate produced from tumour cells also might be converted to glucose by the Cori cycle (futile cycle) in the liver [64, 66], a process that contributes to hypermetabolism and consequently the wasting of host tissue. As demonstrated by a decrease in serum lactate levels for the LW group, probably this futile cycle was minimised in tumour-bearing rats fed a leucine-rich diet and therefore leucine supplementation may improve the host tissue activity minimising the tumour-induced wasting effects.

The tumour-bearing rats also generally showed increases in metabolic pathways that provide nutrient and energy sources for tumour cell growth. The LW group recruited various metabolic pathways (such as ketone body metabolism) that have the potential to increase tumour cell activity (i.e., enhancing Ki-67 and mTOR expression), however, the nutritional supplementation did not appear to benefit tumour growth (Fig. 5). Based on the ^1^H NMR-derived metabolomic profiles, we found that tumour tissue from the LW group had a higher content of lipid deposits in comparison to tumour tissue from the W group. This result suggests a reduction in the ß-oxidation of fatty acids in the Walker 256 cells treated with leucine. In agreement with these results, Shen and colleagues [8] have attributed changes of a metabolomic profile in the urine of Walker 256 tumour-bearing rats to the elevated cell proliferation and reduction in ß-oxidation of fatty acids during cancer progression.

Our results also revealed two potential candidate serum biomarkers for Walker 256 tumour growth, namely 3-methyl-2-oxovaleric acid and 4-hydroxyphenyllactate that were exclusively detected in serum from tumour-bearing groups. The metabolite 3-methyl-2-oxovaleric acid is the alpha-keto acid analogue of isoleucine, produced from isoleucine by cytosolic branched-chain aminotransferase 1, which is a clinical marker of maple syrup urine disease (MSUD) [42]. Notably, until now no research has shown that this metabolite is related to cancer evolution. The other potential candidate biomarker is 4-hydroxyphenyllactate (the L-form), which is a tyrosine metabolite and can be employed to decrease ROS (reactive oxygen species) production in both mitochondria and neutrophils. Therefore, this metabolite might act as a natural antioxidant [42] especially in the tumour-bearing group under a leucine supplementation effect, although again until now no research protocol has related hydroxyphenyllactate with cancer evolution. Furthermore, the high level of sarcosine found in both tumour-bearing groups could indicate the metastatic potential of this Walker 256 tumour, as sarcosine is considered to be an oncometabolite due to its capacity to induce the invasion of cancer cells into other tissues [67].

## Conclusion

Ultimately, a leucine-rich diet implemented to prevent skeletal muscle loss and ameliorate cachexia in Walker 256 cancer rat hosts had no effect on the tumour growth. As determined by solution-phase ^1^H NMR, supplementation did lead to metabolomic alterations that could partially explain the positive effects of leucine supplementation in the rats. In particular, our data suggest that a leucine-rich diet drives metabolomic changes, such as lower levels of tryptophan and lactate (as seen in the LW group), may be associated with a decreased hypermetabolic state and therefore indirectly contribute to minimise the cachexia. Also, the high availability of β-hydroxybutyrate and acetoacetate in the LW group could potentially provide an efficient energy source for skeletal muscle, which also may indirectly contribute to the prevention of cachexia. Although some studies have demonstrated that leucine supplementation may in fact increase cell signalling and tumour cell proliferation [68], here we showed that leucine-rich diet has no effect on tumour evolution. Additional experiments and studies are ongoing in our laboratory to better understand the effect of leucine supplementation on tumour tissue and Walker 256 cell metabolism.
